# Deep Learning-Based Metasurface Design for Smart Cooling of Spacecraft

**DOI:** 10.3390/nano13233073

**Published:** 2023-12-04

**Authors:** Ayman Negm, Mohamed H. Bakr, Matiar M. R. Howlader, Shirook M. Ali

**Affiliations:** 1 Department of Electrical and Computer Engineering, McMaster University, Hamilton, ON L8S 4K1, Canada; negma1@mcmaster.ca; 2 Department of Electronics and Communications Engineering, Cairo University, Giza 12613, Egypt; 3 School of Mechanical and Electrical Engineering Technology, Sheridan College, Brampton, ON L6Y 5H9, Canada; shirook.ali@ieee.org

**Keywords:** CNN, deep learning, metasurface, phase-change, plasmonic, radiative cooling, spacecraft, vanadium dioxide

## Abstract

A reconfigurable metasurface constitutes an important block of future adaptive and smart nanophotonic applications, such as adaptive cooling in spacecraft. In this paper, we introduce a new modeling approach for the fast design of tunable and reconfigurable metasurface structures using a convolutional deep learning network. The metasurface structure is modeled as a multilayer image tensor to model material properties as image maps. We avoid the dimensionality mismatch problem using the operating wavelength as an input to the network. As a case study, we model the response of a reconfigurable absorber that employs the phase transition of vanadium dioxide in the mid-infrared spectrum. The feed-forward model is used as a surrogate model and is subsequently employed within a pattern search optimization process to design a passive adaptive cooling surface leveraging the phase transition of vanadium dioxide. The results indicate that our model delivers an accurate prediction of the metasurface response using a relatively small training dataset. The proposed patterned vanadium dioxide metasurface achieved a 28% saving in coating thickness compared to the literature while maintaining reasonable emissivity contrast at 0.43. Moreover, our design approach was able to overcome the non-uniqueness problem by generating multiple patterns that satisfy the design objectives. The proposed adaptive metasurface can potentially serve as a core block for passive spacecraft cooling applications. We also believe that our design approach can be extended to cover a wider range of applications.

## 1. Introduction

Satellites and orbital artifacts undergo a wide range of temperature fluctuations, exposing them to continuous thermal changes that accelerate equipment wear and spacecraft surface aging [1]. Given the lack of air in space, both heat conduction and convection are inoperative, compelling the spacecraft to rely solely on radiative cooling for the dissipation of excess heat [2]. On the other hand, phenomena such as eclipses can drive the spacecraft surface temperature dangerously low, posing risks to system functionality and necessitating the use of survival heaters [3]. A smart alternative to handle such cases is to design the spacecraft surface with adaptive properties, ensuring heat retention at low temperatures and efficient radiation at high temperatures, a process known as thermal homeostasis [2,4,5]. Energy conservation is critical in spacecraft applications, making power-operated cooling systems impractical. Passive approaches, such as using phase-change materials (PCMs), provide a means of thermal management without the need for external power. Vanadium dioxide (VO_2_) is a volatile PCM that can reversibly transition from an insulator to a metal at 67 °C [6]. Additionally, VO_2_ is characterized by its high thermal capacity during its phase transition [7]. These advantages position VO_2_ as a promising material for various thermal applications in the mid-infrared regime [6,8]. Given that the sun-facing side of the spacecraft can reach temperatures up to 121 °C [9], the phase transition process in VO_2_ can take place passively. To incorporate VO_2_ within the coating layer of the spacecraft, a metasurface structure is appealing due to its subwavelength dimensions and small thickness. This configuration facilitates nanoscale interactions between unit blocks, resulting in optical properties that are unattainable in natural materials [10].

The growing interest in metasurface absorbers can be traced back to the fabrication of the first perfect metasurface absorber in 2008 [11]. Subsequently, these absorbers have been crafted for numerous applications such as energy harvesting, sensing, and imaging [12]. Passive metasurface absorbers have fixed absorption characteristics that cannot be altered after fabrication. Reconfigurable metasurfaces are thus required to compensate for fabrication errors [13], accommodate multiple functionalities [14], and enable response modulation [15]. The deep light-matter interaction of nanoscale metasurfaces, for example, plasmonic effects, has paved the way for a new design frontier where optimized geometries can provide enhanced effects based on their unique plasmonic properties [16]. In the field of thermal control applications, the commonly utilized configuration for plasmonic metasurface structures is the metal–insulator–metal (MIM) arrangement. A metallic reflector is placed at the bottom to block the transmission of radiation, while the spacer layer acts as an interference layer. Typically, the thickness of the spacer is adjusted to λ/4, where λ is the center operating wavelength [17,18]. The unique properties of VO_2_ contribute to the adaptability of the MIM configuration, allowing for dynamic responses to varying thermal conditions. This adaptability is demonstrated in the diverse range of MIM-based absorbers shown in Table 1.

The authors of [25] showed that VO_2_ patches can be employed to construct the adaptive metasurface for passive thermal control of spacecraft. They showed that instead of using conventional VO_2_ thin films, patches could be employed to provide more cost-effectiveness and allow for utilizing plasmonic properties of VO_2_ through the interaction of neighboring patches. The results of that study pointed out that a patterned VO_2_ metasurface can perform better than a thin film. Another study that supports this result was conducted in [5], where a massive change in emissivity between cold and hot states was obtained using an array of Si cones conformally coated with VO_2_. This study showed that using patterned cones provides better contrast than using thin films. However, implementing the cryogenic reactive ion etching approach recommended by the authors presents a difficulty, as it requires maintaining the substrate at extremely low temperatures [26].

The process of metasurface design involves a wide variety of geometries with a broad range of parameter selections, which are tackled using known theoretical models [27], building a library of parameter values [28], or through trial and error. Theoretical models are limited to special use cases [29], and design based on a library of parameters may restrict the exploration of potentially effective parameter ranges. Trial-and-error methods, on the other hand, demand a significant allocation of resources. These limitations urge the need for faster and more efficient approaches for metasurface designs, such as machine learning. Recently, there has been significant interest in employing machine learning for the fast design of tunable and reconfigurable metasurface structures. For example, the authors in [30] designed a reprogrammable metasurface imager using a machine-learning approach where the metasurface was reconfigured using a field-programmable gate array to provide biasing voltages to individual metasurface elements. The authors pointed out that phase-change materials can be a key constituent in future reconfigurable metasurface imagers. Similarly, in [31], a reconfigurable metasurface was employed to design a real-time invisibility cloak with the help of a neural network trained using background and incident wave amplitudes. The concept of a reconfigurable intelligent surface (RIS) was studied in [32], highlighting its considerable potential for deployment in future 6G networks. The inverse design of RIS using different deep learning architectures was reviewed in [33], where the authors showed how deep learning outperforms other inverse design approaches, such as evolutionary algorithms. They also emphasized the potential of deep learning techniques to generate novel designs that might not be achievable through other means. The authors in [34] designed a neural network to predict the EM response of basic geometries such as cylinders and H-shaped metasurfaces. They also demonstrated the use of a combination of feed-forward and inverse deep neural networks for the design of reconfigurable structures given specific operating frequencies and ranges of refractive indices. However, this approach falls short in modeling a hybrid metasurface design that includes multiple materials on the same surface. Moreover, it cannot handle the case of a frequency-dependent refractive index. To design complex geometries, convolutional neural networks (CNNs) have been employed where a metasurface is pixelated and handled as an image. For example, the authors in [35] paired a CNN with a binary particle swarm optimization algorithm to design a binary-coded metasurface for polarization control. In [36], the optical properties of plasmonic structures were analyzed using a CNN applied for binary images. A CNN was also used in [37] to process 1D data of absorption data and output 1D data of geometric parameters. While the existing literature demonstrates the effectiveness of using CNNs to process data from image-like structures, analysis has been constrained to surfaces featuring a single material. This limitation necessitates the retraining networks when introducing different materials.

For the neural network to capture variations in metasurface frequency response over a certain frequency range, it is essential to sample the response space with an adequate number of frequency points. However, there is no specific rule to determine the optimum number of samples. For example, 300 points were used in [38] to span 0.8 to 1.5 THz, 31 points were employed in [34] between 30 and 60 THz, and 32 points were used in [39] to cover the range between 170 and 600 THz. Moreover, as the number of frequency samples increases, the problem of dimensionality mismatch between the input and output parameters worsens, where a limited number of input parameters are mapped to numerous output variables [34,40]. This issue was tackled in [41] using an auto-encoder block [42], wherein both the design space and response space dimensionality were reduced by utilizing a lower dimensional hidden layer in the auto-encoder. A neural network was then configured to model the relationship between the two reduced spaces. The response in the original space can be retrieved by referring to the decoder block of the auto-encoder. This model was used to study the response of a PCM-based reconfigurable gold (Au) structure incorporating germanium-antimony-telluride (GST), whose crystallization level was scaled between 0 and 1. However, modeling GST in this way overlooks the physical significance of the relationship between the permittivity of GST and Au at different phases of GST. Thus, the model cannot be generalized to other cases of material integration. The authors of [40] avoided the mismatch problem using the operating wavelength in its absolute and normalized forms as an input parameter to the network. This way, the network was considered a single-point solver, and so the mismatch problem was avoided.

In this work, we introduce a surrogate-based approach for the design of phase-change and reconfigurable metasurfaces. We employ a CNN to process metasurface structures as 2D images where permittivity maps are used instead of binary encoding utilized in the literature, which provides our network with the capability to model the behavior of new materials by introducing appropriate permittivity values. Moreover, other material properties, such as conductivity, can also be introduced as inputs to the network, which grants more freedom in the material selection of the metasurface structure. The use of dielectric constant maps as input was studied in [43] for the application of a beam-steering metasurface using liquid crystals. Here, we use the permittivity map for the adaptive cooling application of a phase-change metasurface using VO_2_. We show the effectiveness of this design approach using the trained model as a surrogate for the patterned design of the VO_2_-based metasurface. A pattern search algorithm [44] is used to generate the candidate patterns and test their performance in terms of emissivity contrast between hot and cold states of VO_2_. Our approach offers a promising solution for the design of reconfigurable metasurfaces in the infrared regime.

## 2. Materials and Methods

To achieve adaptive thermal control of the spacecraft, its surface must efficiently release excessive heat at high temperatures and maintain heat when the temperature is low. To quantify this performance, the emissivity ε of the structure as a function of the operating temperature *T* is employed [2,4,45]:

(1) $$\varepsilon \left(T\right)=\frac{\int_{\lambda_{min}}^{\lambda_{max}}\left(1-R\left(\lambda \right)\right)B\left(\lambda ,T\right)d\lambda }{\int_{\lambda_{min}}^{\lambda_{max}}B\left(\lambda ,T\right)d\lambda },$$

where *R*(λ) is the wavelength-dependent reflectance of the device and *B*(λ, *T*) is the blackbody radiation function defined by Plank’s law [46,47]:

(2) $$B\left(\lambda ,T\right)=\frac{2hc^{2}}{\lambda^{5}}\frac{1}{e^{\frac{hc}{\lambda KT}}-1},$$

where *h* is Plank’s constant (6.6261 × 10^−34^ J.s), *c* is the speed of light in vacuum (2.9979 × 10^8^ m/s), and *K* is Boltzmann’s constant (1.3806 × 10^−23^ m^2^ kgs^−2^ K^−1^). The main objective is to maximize the emissivity contrast between the two operating states of VO_2_:

(3) $$∆\varepsilon =\varepsilon_{H}-\varepsilon_{L},$$

where $\varepsilon_{H}$ is emissivity measured at 80 °C and $\varepsilon_{L}$ is the emissivity measured at 25 °C. As this contrast increases, the device attains wider modulation capability. Table 2 shows some examples of structures supporting emissivity contrast by utilizing the phase transition of VO_2_. We study the feasibility of using deep learning models to design a pixelated VO_2_ metasurface to achieve high emissivity contrast for adaptive cooling purposes. The proposed structure uses the MIM configuration to block the radiation from escaping the device through transmission. The operating range of the metasurface structure is selected in the mid-infrared range between 2.5 and 25 µm, where the blackbody emission is maximum [2]. We employ a pattern search optimizer to produce multiple solutions using a statistical starting point approach. The optimization algorithm is run multiple times using different starting seed points to explore multiple optimum points in the design space. Therefore, we use the deep network as a surrogate model to avoid non-uniqueness issues. For data generation, we follow an incremental approach guided by the output of the pattern search optimizer to avoid over-sampling the parameter space in less critical regions.

### 2.1. Structure and Data Generation

**Figure 1:**
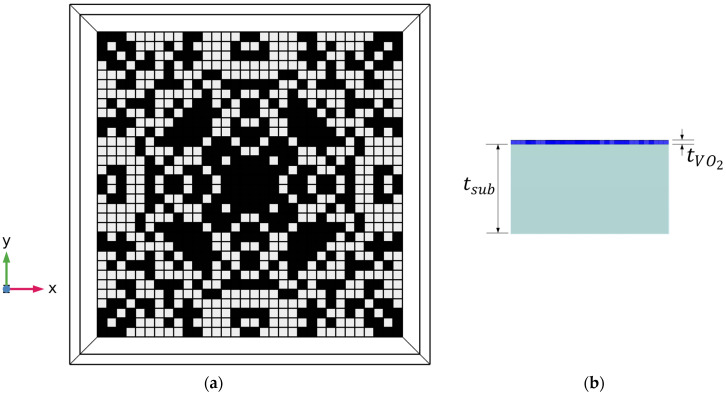
(**a**) Top and (**b**) side views of one VO_2_-based pixelated structure generated using COMSOL (black pixels stand for VO_2_ patches, white pixels stand for void pixels). Quarter symmetry is imposed to ensure polarization-independent performance. t_VO2_ is fixed at 40 nm, while t_sub_ is used as an input variable to the modeling network.

Figure 1 shows the top and side views of the structure studied in this work. A MIM configuration is employed with a fixed bottom metallic reflector that is thick enough to block the transmission. The top layer is a pixelated layer of VO_2_ patches, each dimension 100 nm × 100 nm. We limit our study to a fixed VO_2_ thickness of 40 nm to speed up the phase transition process [52]. The period of the metasurface is fixed at 3.2 µm, allowing for VO_2_ patterns of 32 × 32 pixels. The quarter symmetry is maintained for all the generated patterns to achieve polarization-independent behavior. This means that the actual design space is only 16 × 16 for one quarter, which is then copied by symmetry to the other three quarters. The choice of the substrate depends on the required properties within the operating band and the target application. For spacecraft applications, it is desired to have a substrate that is easy to fabricate and pattern. The authors in [25] recommend using a low-emissivity substrate such as CaF_2_ or MgF_2_. However, the fabrication process of fluorides is more complicated than that of oxides [18]. Moreover, fluoride substrates are subject to oxidation under the effect of the abundant atomic oxygen in space, which degrades their performance [53,54]. For these reasons, we choose silicon dioxide as the substrate for our structure, which can provide reasonable emissivity in the infrared range of 4.5–25 µm [55]. Since the plasmonic interactions of VO_2_ pixels vary with the distribution of pixels, we show in our study that the optimum thickness can go much lower than this value, which provides a cost-effective design for the spacecraft surface. For this reason, the thickness of the substrate is employed as an input parameter to the deep learning network, together with the VO_2_ pattern and the operating wavelength.

We use COMSOL Multiphysics 6.0 [56] to generate the reflection data *R*(λ) to evaluate the emissivity as defined in Equation (1). The structure is excited by a linearly polarized plane wave, with periodic boundary conditions imposed on the lateral sides, and perfectly matched layers applied to the top and bottom to eliminate undesired reflections. The values of the refractive index data for VO_2_ at cold and hot states are obtained from the experimental measurements reported in [57].

**Figure 2:**
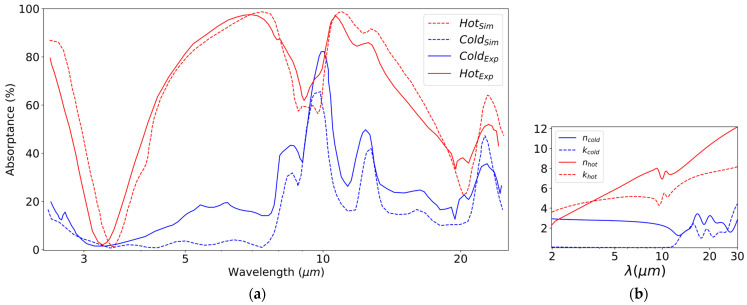
(**a**) Verifying the accuracy of the COMSOL model against experimental data in [24]. (**b**) The refractive index of VO_2_ used in simulations.

We verify the accuracy of our COMSOL solver by regenerating the experimental data reported in [24] (see Figure 2a). The root-mean-squared error between the model and experimental data is 4.9% for the hot state and 11.5% for the cold state. The authors of [24] reported [25] that annealing temperature can affect response in the cold states, which means that the error can go even lower if a higher annealing temperature is employed. Figure 2b shows that the extinction coefficient (k) of VO_2_ is very small in the cold state, leading to very low absorption of VO_2_ in this cold state. Consequently, the absorption modes are dominated by those of the SiO_2_ substrate [25,58].

**Figure 3:**
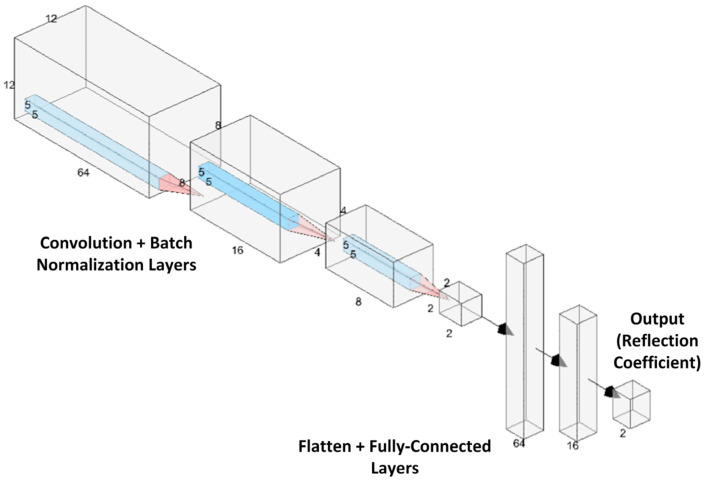
Proposed deep learning architecture for modeling the response of pixelated VO_2_ metasurface.

To quickly predict the response data *R*(λ) given an input configuration, we choose a CNN as the modeling structure because of its powerful ability to process image-like data. Figure 3 shows the structure of the deep CNN used for modeling the metasurface response. The structure employs convolutional filtering stages starting with 64 filters in the first layer, followed by 16, 8, and then 2. Each convolutional stage is followed by a batch normalization step to remove the biasing effect in data [37]. In addition, dropout layers with a 40% ratio are used to improve the network’s ability to generalize the network to unseen data. The output of these layers is then combined with the scalar wavelength and substrate thickness inputs and introduced to a set of fully connected layers of 64, 16, and 2 neurons, respectively. Since *R*(λ) is a complex variable, we define the outputs of the CNN to be the real and imaginary parts. The training of the network parameters is performed using a stochastic gradient descent algorithm [59], with a learning rate of 0.01 and momentum of 0.9. Random sampling techniques, such as latin hypercube sampling, can be used to generate training patterns [60]. However, random sampling may not be the optimal data generation approach as the network may require non-linear sampling of the parameter space. A similar approach was used in [61] to specify the size and orientation of a metasurface patch. The surrogate approach was also used in [62,63], but an encoder was used to speed up optimization and limit the design search space without sacrificing accuracy.

Since we are interested in the behavior of the metasurface structure in the mid-infrared band between 2.5 and 25 µm, the forward model should be able to predict the response by employing data generated within this range. The approach followed in the literature for this purpose is to arbitrarily define several wavelength points to generate the data for and to capture the peaks and valleys of the response. The number of samples is considered sufficient when all the response variations are captured. However, there is no specific rule on how to choose the number of samples, and it is selected by trial and error. In addition, some recurrent models employ the spectrum as a sequence of data to predict the corresponding geometrical parameters [64].

The authors of [40] showed that the operating wavelength (or frequency) can be used as input to the network to overcome the sampling issue. This approach avoids the burden of capturing the response variations, which are automatically learned as the deep network operates as a single-wavelength solver. In addition, this approach also avoids the dimensionality mismatch challenge highlighted in [65], where a small number of design parameters (e.g., dimensions of the nanostructure, substrate thickness, etc.) are used to predict a large number of output features, which are the spectral response samples. Using the wavelength as input, the mismatch problem can be completely avoided.

### 2.2. Inverse Problem

A major challenge in solving the inverse problem is the non-uniqueness issue, where different metasurface patterns can produce the same response [66], which renders the problem a one-to-many mapping. To avoid this issue, methods reported in [67] include filtering the training dataset, where designs that produce nearly the same response are filtered such that only one of them is saved in the dataset. Another method is to divide the training space into sub-spaces such that the problem is one-to-one in each of them [68], but these methods have limited effectiveness. Performance can be improved by constructing two deep networks: one to model the forward model and the other to model inverse mapping, a structure that is known as the tandem network [69]. The feed-forward network is used as a full-wave solver that is used to generate the response, while the inverse network is fed by the output of the feed-forward network. The output response is then compared against the original desired response to guide the training process of the inverse network. This way, the solution always converges even if there are non-unique samples in the training set. However, this approach will provide only one design corresponding to each desired response, even though multiple designs can produce the given response, as highlighted in [70,71]. The authors in [70,71] proposed using Gaussian mixtures as an alternative to neural networks to model the design parameters in terms of Gaussian distribution parameters. The specific design that the network will produce will depend on the weights of the network. A very similar approach was followed in [72], where the inverse network was modeled as a general adversarial network (GAN) network, but the tandem concept was the same. We can easily see that this will not show us the different designs that can produce the response. In addition, the tandem network requires two separate training processes, which may be complicated.

To provide the network with the ability to generate multiple designs for a given response, the authors in [41] proposed the use of auto-encoders, where instead of using the original design space and response space as inputs and outputs to the deep network, the auto-encoder intermediate block was used to reduce the dimensionality of both spaces. The mapping between the reduced design space and reduced response space was one-to-one, which was easily learned by the deep network. A close approach was adopted in [73], where the authors employed a probabilistic model to relate the design parameters to latent variables space, and multiple designs were obtained by sampling the latent space. Although these approaches provide a way to generate multiple patterns for a given response, the response space is still sampled at an arbitrary number of points, which will affect the accuracy of the mapping. In addition, building multiple deep networks is involved, which may complicate the training process.

To avoid this complexity, a surrogate-based approach can be used where only one network is trained to model the forward behavior of the structure, and then it is used within an optimization algorithm to complete the inverse design process [61,62,63]. In our study, we employ a CNN as a forward-problem solver or a surrogate model, and we use a pattern search optimizer to obtain the optimized structures. We also implement active sampling, as demonstrated in [60], to design the sought adaptive VO_2_-based metasurface. To avoid sampling the response space at an arbitrary number of points, we adopt the approach followed in [40], where we use the operating wavelength as an input to the forward solver and find the corresponding response, which means that the dimension of the response space becomes 1.

To determine the global optimum design that would maximize the emissivity contrast as defined in Equation (3), we employ the pattern search algorithm. This algorithm was developed by Hooke and Jeeves in 1960 [44]. The algorithm follows a derivative-free, direct search approach to reach the optimum by defining the current search direction based on the history of iterations. The algorithm employs two types of moves: exploration and pattern. In the exploratory step, each coordinate of the solution space is varied while retaining all the other fixed coordinates. The process continues until all coordinates are explored for the optimum point. In the pattern step, the algorithm predicts the next starting point by observing the pattern of the motion towards the global optimum. The algorithm switches between these two steps until a stopping criterion is met. The selection of this algorithm for our design problem is mainly for two reasons. The first one is simplicity; as this algorithm is a direct search approach, it requires minimum settings for parameters and tuning. Second, the algorithm works on observing the effect of each optimization coordinate sequentially. This is translated to tuning pixels of our metasurface pixel by pixel until the optimum pattern is obtained. We use the algorithm implemented in the Pymoo package written using Python 3.12.0 [74].

## 3. Results and Discussion

**Figure 4:**
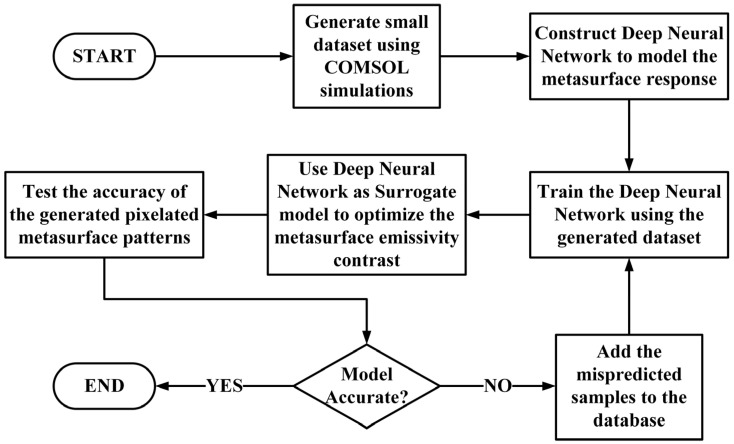
Flow chart of the adaptive metasurface design process employed in this study.

Figure 4 shows the flow chart of the pixelated metasurface design approach. The dataset was initialized by running 10 COMSOL simulations, and then the deep learning network was trained for 100 epochs. At the early training stage, the deep network did not have enough data to generalize input–output mapping but it was still used to develop some proposed patterns based on the most recent values of the network weights. The responses corresponding to the generated patterns were calculated to verify that the predicted emissivity matched the simulated one. The decision to add a pattern to the database was based on the mean-squared error between the simulated and predicted responses. The simulated samples were sequentially added to the database until the error was less than 0.05. Using this active sampling technique, we avoided generating a large dataset and the solution was obtained faster. The training of the network was performed repeatedly to update the weights accordingly. The process continued in an iterative way until the response of patterns generated by the optimizer matched those generated using actual COMSOL simulations.

**Figure 5:**
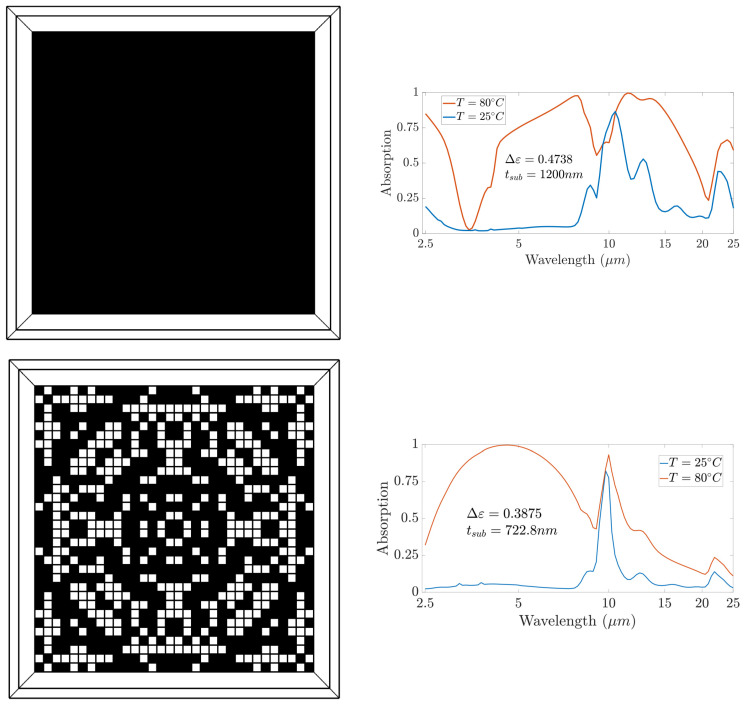

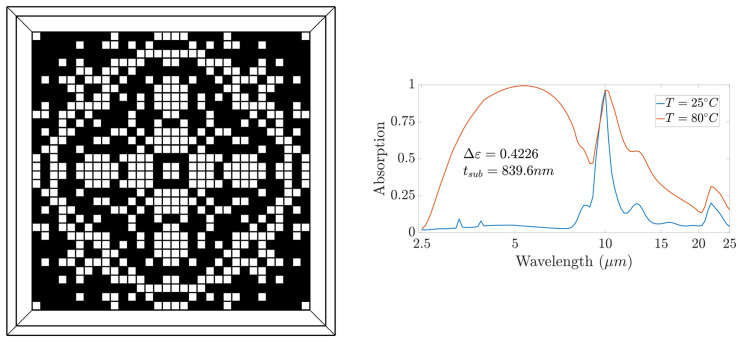
VO_2_ patterns (**left**) and the corresponding absorption behavior at hot and cold states (**right**) of patch metasurface, designs 1 and 2. Black spots represent VO_2_ pixels, while white spots represent void pixels.

**Figure 6:**
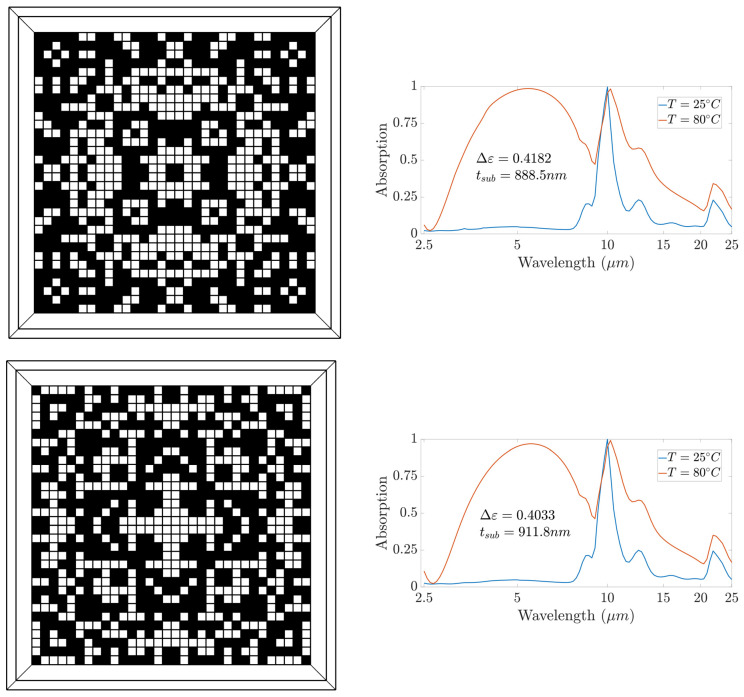

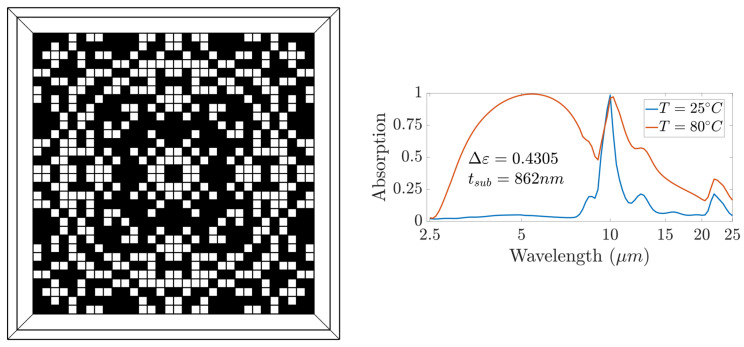
VO_2_ patterns (**left**) and the corresponding absorption behavior at hot and cold states (**right**) for designs 3, 4, and 5. Black spots represent VO_2_ pixels, while white spots represent void pixels.

The final deep learning model achieved a validation mean-squared error of 0.0267 using only 6400 dataset samples. Given that the design space covers 256 pixels, i.e., a total design space of 2^256^, the approach provides an efficient procedure for accurate modeling using a very small dataset. The trained model was used to determine the optimum distribution for the placement of VO_2_ pixels, where the optimality is defined in terms of maximizing the emissivity contrast over the infrared band between 2.5 µm and 25 µm. Without loss of generality, we set the optimizer to have five different starting points corresponding to five generated patterns. Figure 5 and Figure 6 show the five generated patterns with the corresponding responses. We observed that the substrate thickness in our optimal designs was around 862 nm. This represents a 28% reduction compared to the 1200 nm thickness reported in the related literature [25], which is crucial for minimizing the weight and cost of the spacecraft coating. These outcomes align with the results reported in [75], where the authors showed that a patterned metasurface provides improvements in thickness, weight, and cost. Moreover, the bottleneck of the design was the phonon absorption modes of the silica substrate, which were present in both hot and cold states of VO_2_. The effect of these modes increased when the thickness of the substrate increased; therefore, the decreased substrate thickness in our proposed designs is a big advantage. A possible solution to overcome this problem is to use alternative materials that do not suffer from phonon bands, such as BaF_2_ and CaF_2_, taking into consideration the challenges in fabricating this type of fluoride.

**Figure 7:**
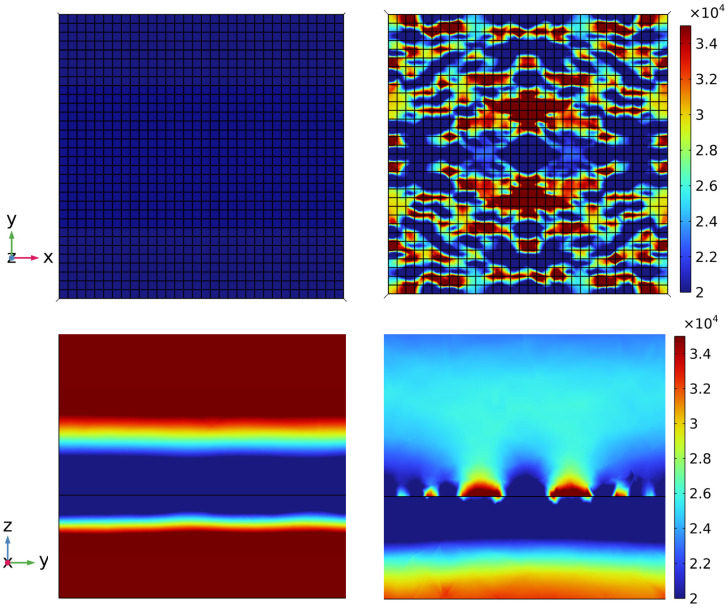
Horizontal (**top**) and vertical (**bottom**) magnetic field distribution (in A/m) for design 5 at the cold (**left**) and hot (**right**) states of VO_2_.

The results also show that the patterned metasurface achieved higher absorption for the hot state than the thin-film design in the range of 3–9 µm at the expense of lower absorption in the range of 12–20 µm. To gain more insight into the absorption behavior of our proposed structure, we plotted the magnetic field distribution of design 5 at 5 µm for the cold and hot states of VO_2_ (see Figure 7). The results indicate that in the cold state, the pixels did not exhibit significant absorption, and absorption was dominated by the substrate. In the hot state, the VO_2_ pixels attained metallic properties, and absorption was dominated by the field concentrated within the metallic pixels of the pattern. We also observed the similarities in the responses of the five patterns, which confirmed that our design approach can overcome the non-uniqueness problem by generating multiple patterns that achieve closely similar responses. This capability is not feasible in widely used models such as tandem networks, which can only generate one pattern corresponding to each desired response based on the training data provided. We generated five samples to demonstrate the ability of our approach to generate multiple patterns using five different initial solutions. The number of possible designs was not limited to five and could increased by expanding the number of initial solution candidates for the optimizer. A specific design could then be chosen based on fabrication constraints and complexity.

Considering the computational aspects of the optimization performed, we showed how the deep-learning approach proposed provides a faster and more reliable solution compared to conventional optimization approaches such as genetic algorithms. Assuming 100 wavelength points are used to calculate the emissivity contrast for a proposed solution and given that one wavelength requires 1 min to simulate, a solution candidate in an evolutionary algorithm will require 100 min to calculate the objective. For a population of 20 candidates, this translates to 2000 min per iteration. If it is required to run 100 iterations to reach an appropriate solution, this means that 200,000 min or 139 days are required to complete the optimization process. On the other hand, the deep learning approach we proposed starts by generating a dataset using 100 simulations, which require 100 min to finish. The deep neural network training takes around 5 min to finish. This trained network is then used as a surrogate model for the optimizer to determine a proper design, which takes around 15 min. To verify the network accuracy, three simulations at three randomly selected wavelengths are performed after optimization to verify the match between the response of the solution candidate and the response of the COMSOL simulation, which takes 3 min. The overall time for a single iteration is 23 min. Assuming the network provides the correct design in 300 iterations, the deep learning approach requires 7000 min or 5 days to reach the optimum result. This means that the deep learning approach reduces the optimization time by a factor of 27.8, which is a significant saving that can be even more crucial for structures that require a longer time to simulate.

## 4. Conclusions

In this work, we introduced a new modeling approach for reconfigurable metasurfaces using a convolutional neural network. Material properties were represented as image tensors and fed into the network as inputs together with the operating wavelength. We prevented the mismatch problem by associating a single output specifically with the input wavelength. The model can be used to include more material properties by simply adding more image layers to the input tensor. The model can also be used to predict the response of geometries with hybrid material composition and phase-change metasurfaces with multiple crystallization levels. This modeling approach simplifies the design and analysis of hybrid and reconfigurable metasurface structures. We employed a deep neural network as a surrogate model together with a direct search optimizer to obtain multiple configurations for a pixelated VO_2_ metasurface that can provide emissivity contrast for adaptive thermal control of a spacecraft. Our deep learning approach provides an efficient data generation procedure and adeptly addresses the non-uniqueness problem inherent in inverse solutions.

## Figures and Tables

**Table 1:** Examples of MIM structures employing VO_2_.

VO_2_ Structure	Substrate	Reflector	Operating Range (µm)	Ref.
Patches	Si	Tungsten (W)	4–14	[19]
Thin film	N/A	Al	5–25	[20]
Thin film	Si	Al	5–20	[21]
Thin film	BaF_2_	Au	5–25	[2]
Thin film	MgF_2_	W	4–14	[22]
Trapezoidal Multi-layer	MgF_2_ + Ge	Ti	5–14	[23]
Cones	Si	Au	2.5–30	[5]
Thin film	Si	Au	5–30	[4]
Patches	SiO_2_	Al	2.5–20	[24]

**Table 2:** Examples of adaptive structures employing VO_2_ for emissivity contrast.

VO_2_ Structure	Substrate	Reflector	Operating Range (µm)	$\varepsilon_{L}$	$\varepsilon_{H}$	Δε	Ref.
Thin film (30 nm)	SiO_2_	Au	2.5–25	0.22	0.71	0.49	[48]
Thin film (40 nm)	BaF_2_	Au	5–25	0.16	0.51	0.35	[2]
Patches (40 nm)	SiO_2_	AZO	2.5–20	0.54	0.81	0.26	[25]
Thin film (50 nm)	Al_2_O_3_	Ag	5–15	0.34	0.87	0.53	[45]
Thin film (50 nm)	HfO_2_	Al	2.5–25	0.23	0.74	0.51	[49]
Thin film (60 nm)	Si	Al	5–25	0.14	0.6	0.45	[20]
Thin film (62 nm)	Si	Au	2–30	0.22	0.46	0.24	[4]
Thin film (263 nm)	SiO_2_	Al	5–25	0.18	0.57	0.39	[50]
Thin film (360 nm)	SiO_2_	Ag	5–20	0.07	0.59	0.52	[51]

## Data Availability

Data underlying the results presented in this paper are not publicly available at this time but may be obtained from the authors upon reasonable request.
